# Psychosocial work environment factors and weight change: a prospective study among Danish health care workers

**DOI:** 10.1186/1471-2458-13-43

**Published:** 2013-01-17

**Authors:** Helle Gram Quist, Ulla Christensen, Karl Bang Christensen, Birgit Aust, Vilhelm Borg, Jakob B Bjorner

**Affiliations:** 1 National Research Centre for the Working Environment (NRCWE), Lersoe Parkalle 105, 2100, Copenhagen, Denmark; 2 Department of Public Health, Section of Social Medicine, University of Copenhagen, Oester Farigmagsgade 5, 1014, Copenhagen, Denmark; 3 Department of Public Health, Section of Biostatistics, University of Copenhagen, Oester Farigmagsgade 5, 1014, Copenhagen, Denmark; 4 QualityMetric/OptumInsight, 24 Albion Road, Lincoln, RI, 02865, USA

**Keywords:** Body mass index, Weight gain, Weight loss, Work stress, Psychosocial work factors

## Abstract

**Background:**

Lifestyle variables may serve as important intermediate factors between psychosocial work environment and health outcomes. Previous studies, focussing on work stress models have shown mixed and weak results in relation to weight change. This study aims to investigate psychosocial factors outside the classical work stress models as potential predictors of change in body mass index (BMI) in a population of health care workers.

**Methods:**

A cohort study, with three years follow-up, was conducted among Danish health care workers (3982 women and 152 men). Logistic regression analyses examined change in BMI (more than +/− 2 kg/m^2^) as predicted by baseline psychosocial work factors (work pace, workload, quality of leadership, influence at work, meaning of work, predictability, commitment, role clarity, and role conflicts) and five covariates (age, cohabitation, physical work demands, type of work position and seniority).

**Results:**

Among women, high role conflicts predicted weight gain, while high role clarity predicted both weight gain and weight loss. Living alone also predicted weight gain among women, while older age decreased the odds of weight gain. High leadership quality predicted weight loss among men. Associations were generally weak, with the exception of quality of leadership, age, and cohabitation.

**Conclusion:**

This study of a single occupational group suggested a few new risk factors for weight change outside the traditional work stress models.

## Background

Obesity is a well-known risk factor for mortality and morbidity, including cardiovascular disease, hypertension, stroke, type II diabetes and some types of cancer [1-3]. The prevalence of obesity has grown worldwide, reaching epidemic proportions. Previous research has found that weight change, both loss and gain, carries a risk of mortality independently of the initial weight level; and that the risk of mortality from weight change is higher than the risk from a stable weight [4]. Maintaining a stable weight seems favourable when addressing the risk of mortality.

Body weight is not just a simple matter of energy balance (calorie intake and physical activity), but also a matter of genetic and socioeconomic factors [5,6]. Evidence shows that psychosocial work factors affect our health [7-13] and health behaviour, such as physical activity, drinking, smoking and dietary habits [14-19]. These health behaviours may be intermediate factors in the relationship between psychosocial work environment and health related outcomes. Longitudinal research studies on working conditions and weight change are scarce and have mainly looked at traditional work stress models, particularly the job-strain model [12] and the effort-reward imbalance model [20]. In both models, it is assumed that work stress can alter our health behaviours, causing - for instance - weight change. Overall, results from previous studies are inconsistent. Where some studies have found full support for a positive association between poor psychosocial working conditions and weight gain [8,21-23], others have found no consistent support [24-26], a bi-directional relationship [27,28], or that poor psychosocial working conditions are associated with weight loss [29-31]. Also, research suggests that stress can cause some people to eat more, while others react by eating less [32,33]. Epidemiological researchers have also tied body weight/weight change to other work and sociocultural factors, such as shift work, working overtime, education, income and marital status [34-39]. Again results are mixed and vary between genders.

As the job-strain model [12] and the effort-reward imbalance model [20] have shown inconsistent results, it is worth broadening the area of psychosocial work environment, as it is possible that other psychosocial work factors could have an effect on weight change. This was done in a recent study by Berset and colleagues, who examined the role of social stressors on the relationship between weight gain and work stress [40]. They found that social stressors, such as tension between colleagues and the use of reprimands, were predictive of BMI at follow-up, while only finding limited support for the role of work stress. This adds to the idea that other aspects of the psychosocial work environment might be of bigger importance than previously assumed. Consequently, this explorative study uses a broader mapping of the psychosocial work environment, based on the Copenhagen Psychosocial Questionnaire (COPSOQ) [41]. The COPSOQ is theoretically founded but not restricted to a single theory [42] and is developed on the basis of existing questionnaires, such as the Setterlind Stress Profile [43], the Whitehall II questionnaire [44], the Job-Content Questionnaire [45] and Short Form-36 [46].

In addition to the dimensions from the job-strain and effort-reward imbalance models, the COPSOQ includes other dimensions from the psychosocial area, such as emotional demands, cognitive demands, meaning of work, quality of leadership, predictability, role conflicts, social support and offensive behaviours. In a recent study, high emotional demands and low meaning of work, was shown to predict ill mental health beyond the job strain and effort-reward imbalance models [47]. Further, role conflicts, high emotional demands, low quality of leadership and the demands for hiding emotions, have been found to predict sickness absence in Danish populations [48,49]. Using dimensions from the COPSOQ, the aim of the present study is to examine the relationship between a broad range of psychosocial work factors and change in BMI. We see weight change as an indicator of lifestyle and health behaviour. We examined change in BMI bi-directionally; both as an increase and decrease. We hypothesize that an unfavourable psychosocial work environment (e.g. low meaning of work, low predictability etc.) is associated with change in BMI. Our analyses take into account that work environment stressors can increase the risk of both weight gain and weight loss as individual responses to stress may vary.

## Methods

### Study design and study sample

We used data from a prospective cohort study of employees in the eldercare sector in Denmark. The study aims at examining the health and work environment of health care workers employed in Danish municipalities (36 out of 65 invited municipalities participated). Baseline questionnaire data was collected from fall 2004 to spring 2005 using mailed questionnaires. These were distributed to 12,746 employees at baseline – of which 9,949 participated (78%). A follow-up was conducted in 2006, preceding a second follow-up in 2008. Participation in the follow-up rounds was 64% and 63%, respectively. Maximizing the follow-up period to three years, this study only included respondents who participated at baseline and in the second follow-up round (3982 women and 153 men). Table 1 presents descriptive characteristics of the participants at baseline. An analysis of difference between the study population (those responding to both baseline and follow-up) and those only examined at baseline, showed no significant differences with regards to age, body mass index and leisure-time physical activity. However, the study population had significant longer tenure (mean 8.9 vs. 8.0 years, p < .0001), lower physical demands at work (mean 18.6 vs. 19.6, p < .0001) and had a lower proportion of smokers (34% vs 36%, p = 0.0151). With regards to the psychosocial work environment, we found that the study population reported higher predictability (mean 58 vs 56, p < .0001), higher influence (mean 47 vs 45, p < .0001), higher leadership quality (mean 59 vs. 56, p < .0001), higher involvement (mean 60 vs. 58, p < .0001), higher meaning at work (mean 79 vs. 77, p = 0.0006), but lower role conflicts (mean 41 vs. 42, p = 0.0133). Thus, it seems that the study population experience a better psychosocial and physical work environment that the sample only examined at baseline. However, the differences in work environment appear to be small.

**Table 1:** Baseline descriptive statistics of the study population

	**Men**				**Women**			
**Variable**	**N**	**%**	**Mean**	**S.D**	**N**	**%**	**Mean**	**S.D**
*Baseline 2004/2005*								
Age	153		46	8.25	3982		45	8.45
Smoke status								
Smoker	32	20.92	.	.	1389	35.18	.	.
Non-smoker^a^	121	79.08	.	.	2559	64.82	.	.
BMI (kg/m^2^)	151		25.97	3.30	3804		24.89	4.34
Cohabitation								
Living with a partner or spouse	122	80.79	.	.	3245	82.38	.	.
Living alone/other	29	19.21	.	.	694	17.62	.	.
Work Hours								
Day schedule	122	79.74	.	.	2658	66.87	.	.
Fixed evening shift	17	11.11	.	.	581	14.62	.	.
Fixed night shift	5	3.27	.	.	181	4.55	.	.
Alternating day/evening shift	8	5.23	.	.	441	11.09	.	.
Alternating evening/night shift	0	0.00	.	.	27	0.68	.	.
Alternating day/evening/night shift	1	0.65	.	.	87	2.19	.	.
Type of work position								
Managers	45	29.41	.	.	267	6.71	.	.
Health care workers	73	47.71	.	.	3378	84.83	.	.
Other (eg. janitors, secretaries)	35	22.88	.	.	337	8.46	.	.
*Follow-up 2008*								
BMI (kg/m^2^)	150		26.21	3.24	3776		25.37	4.62
Change in BMI								
Unchanged weight	125	83.89	.	.	2853	77.72	.	.
BMI gain of 2 kg/m^2^	16	10.74	.	.	583	15.88	.	.
BMI loss of 2 kg/m^2^	8	5.37	.	.	235	6.40	.	.

^a ^including previous smokers.

The study was approved by the Danish Data Protection Agency and followed the regulations for data storage and protection. Questionnaire research in Denmark does not require approval by ethic committees and thus approval was not obtained. Before completing the questionnaire, participants were informed about the study and it was made clear that participation was voluntary. Participants returned the completed questionnaires directly to the research group and confidentiality was maintained by using numbers to identify participants.

### Measurements

#### Psychosocial work characteristics

The psychosocial work environment was examined with items and scales derived from the 1^st^ version of the Copenhagen Psychosocial Questionnaire (COPSOQ) [41,50]. We measured the following psychosocial work environment factors: *quantitative demands, quality of leadership, influence at work, meaning of work, commitment, predictability, role clarity and role conflicts*. These are recognized in the literature as important psychosocial work exposures and have been used in other studies [7,10,47,49,51,52]. *Quantitative demands* were measured by a single-item regarding work pace and a two-item scale about workload. *Quality of leadership* was measured by a four-item scale about the managements’ ability to plan work, solve conflicts and ensure well-being and development opportunities for their employees. *Influence at work* was measured by four questions related to personal influence on tasks, amount of work and choice of co-workers. *Meaning of work* was measured by a three-item scale about the meaningfulness and importance of work, work motivation and involvement. *Commitment* to the workplace was measured by a five-item scale concerning personal dedication to the workplace. *Predictability* was measured by a two-item scale about information related to organizational changes and information required to carry out ones job well. *Role clarity* was measured by a three-item scale regarding expectations, objective and responsibilities at work. Finally, *role conflicts* were measured by a four-item scale about the experience and acceptance of tasks, contradictory demands and unnecessary tasks. All psychosocial scales were re-coded from 0 to 100 points, with low scores indicating low levels of the measured dimension. Depending on the scale, a low score can be positive (e.g. low role conflicts) or negative (e.g. low leadership quality).

### Working hours

As working hours are related to psychosocial work environment, e.g. demands and influence [53], we also analyzed working hours. Working hours were divided into two categories: day schedule (fixed dayshift) and non-day schedule (fixed evening shift, fixed night shift, alternating day/evening shifts, alternating evening/night shift or alternating day/evening/night shift). We collapsed all the non-day schedule groups into one group, as some of them only had very few participants (see Table 1).

### Covariates

The covariates in this study were age, cohabitation, type of work position, seniority and physical work demands. Cohabitation was dichotomized as living alone or living with a spouse or partner. Type of work position included managers, health care workers, or others (e.g. janitors, cleaners, secretaries). Seniority specified the number of years respondents had worked at their current workplace. Finally, physical work demands were calculated as a single mean score based on 15 questions regarding lifting, position of the arms, and work postures (for example, “How often does your work require that you kneel down; on one or both knees?” or “How often do you work with a rotated upper body?”) [54]. Respondents were asked how often they were exposed to these positions and lifts (response categories ranged from “never” to “very often”). The higher the mean score, the more demanding physical work requirements.

### Outcome

Body mass index was calculated based on the respondents’ self-reported information on height and weight (weight in kilograms divided by height in meters squared). We measured change in BMI (gain or loss) of more than 2 kg/m^2^. Change in BMI was separated into the following three groups: (1) unchanged weight, (2) BMI loss of more than 2 kg/m^2^, and (3) BMI gain of more than 2 kg/m^2^. Unchanged weight was the reference group. To our knowledge, there is no commonly accepted standard as to what constitutes a meaningful weight change over time. Research indicates that it is desirable to maintain a stable weight (in terms of mortality risk) as almost any level of weight change is associated with increased risk of mortality compared to maintaining a stable weight [4]. To achieve a sufficient group size for a robust analysis, we chose a cut-off point at +/− 2 kg/m^2^ as almost 28% of the respondents had gained/lost more than 2 kg/m^2^, while only 10% gained/lost more than 5 kg/m^2^.

### Statistical analysis

Using logistic regression for nominal categorical data, we assessed the associations between psychosocial work factors and weight change. Specifically, change in BMI was utilized as the dependent variable and psychosocial work factors as predictors. The bi-directional approach allowed us to determine whether psychosocial work factors can lead to increased BMI, decreased BMI or both. We conducted both unadjusted and adjusted regression analyses. In the adjusted model, a backward elimination approach was utilized, starting with all variables included to mutually adjust for each predictor variable. The variables were tested for statistical significance each at the time, deleting those that were not significant. Variables with a significance level greater than the criterion level of 0.05 were removed from the model (the least significant first). All psychosocial variables, in addition to seniority and physical work demands, were included as continuous variables in the statistical analyses. The psychosocial work environment factors were all assessed as continuous variables (originally scores from 0 to 100). However, we changed scoring to 10-points scales (0 to 10), so that the OR represents a 10-point increase in the psychosocial variable. Also, age and physical workload were changed to represent a 10-year and 10-point increase, respectively. All analyses were conducted separately for men and women. The statistical analyses were performed with SAS Proc Logistics, version 9.2 (SAS Institute).

Since we collapsed all non-day schedule workers into one group, we tested whether any loss of information was caused by this day/non-day dichotomization. Furthermore, in order to evaluate possible loss of information due to the categorization of BMI change, we also performed additional analyses using BMI as a continuous variable (using SAS Proc Mixed with logarithmic transformation of BMI to achieve model fit). In addition, we also conducted sensitivity analyses; we tested whether the association between change in BMI and the psychosocial work factors were similar regardless of the work factors being treated as continuous variable or categorical variables (Proc Logistic, SAS, version 9.2). Finally, we checked for multicollinearity among the predictor variables (Proc Reg, SAS, version 9.2).

## Results

Table 1 presents sample characteristics for women (n = 3982) and men (n = 153). On average, the women were younger and had slightly lower mean BMI at baseline than men. In both sexes, BMI increased over the 3-year follow-up period (0.25 kg/m^2^ for men and 0.48 kg/m^2^ for women). More women than men were current smokers (35% and 21%, respectively) and more than 8 out of 10 lived with a spouse or a partner (81% and 82%, for men and women respectively).

A total of 3647 women and 136 men were included in the regression analyses as 335 women and 17 men were excluded due to missing values on the response or explanatory variables. In Table 2 the unadjusted and adjusted estimates are reported for the association between each predictor and change in BMI (odds ratio and 95% confidence intervals are presented). Among men, high leadership quality (OR = 1.55; 95% CI: 1.02-2.36) increased the odds of weight loss. For women, high role conflicts increased the odds of weight gain (OR = 1.13; 95% CI: 1.06-1.21). High role clarity increased the odds for both weight gain (OR = 1.09; 95% CI: 1.02-1.17) and weight loss (OR = 1.14; 95% CI: 1.03-1.27) among women. In addition to the psychosocial work factors, we also found that living alone (OR = 1.33; 95% CI: 1.06-1.68) increased the odds for weight gain, while older age significantly decreased the odds for weight gain (OR = 0.71; 95% CI: 0.64-0.79). Except for age, cohabitation, and quality of leadership all associations were weak.

**Table 2:** Logistics regression with BMI change as dependent variable

		**Women**				**Men**			
		**Unadjusted**		**Mutually adjusted**^**a**^		**Unadjusted**		**Mutually adjusted**^**a**^	
Variable	Response	OR	95% CI	OR	95% CI	OR	95% CI	OR	95% CI
Quality of leadership	BMI gain	0.98	0.94-1.02	.	.	1.27	0.96-1.69	1.29	0.96-1.73
	BMI loss	1.00	0.94-1.07	.	.	**1.48**	**1.00-2.19**	**1.55**	**1.02-2.36**
Influence at work	BMI gain	1.01	0.96-1.05	.	.	1.17	0.90-1.52	.	.
	BMI loss	0.99	0.93-1.06	.	.	1.14	0.77-1.67	.	.
Meaning of work	BMI gain	1.02	0.95-1.09	.	.	1.10	0.75-1.62	.	.
	BMI loss	1.07	0.97-1.19	.	.	**1.95**	**1.04-3.66**	.	.
Workload	BMI gain	0.99	0.95-1.04	.	.	1.03	0.79-1.34	.	.
	BMI loss	0.97	0.91-1.03	.	.	0.75	0.51-1.12	.	.
Work pace	BMI gain	1.01	0.97-1.06	.	.	1.19	0.89-1.59	.	.
	BMI loss	0.94	0.87-1.01	.	.	0.80	0.53-1.20	.	.
Commitment	BMI gain	0.97	0.92-1.02	.	.	1.07	0.78-1.45	.	.
	BMI loss	0.95	0.88-1.03	.	.	1.63	0.98-2.71	.	.
Role clarity	BMI gain	1.03	0.97-1.10	**1.09**	**1.02-1.17**	0.99	0.71-1.38	.	.
	BMI loss	**1.11**	**1.00-1.22**	**1.14**	**1.03-1.27**	**1.97**	**1.03-3.79**	.	.
Role conflicts	BMI gain	**1.13**	**1.06-1.19**	**1.13**	**1.06-1.21**	1.00	0.74-1.35	.	.
	BMI loss	1.02	0.93-1.11	1.04	0.94-1.14	0.84	0.54-1.31	.	.
Predictability	BMI gain	0.97	0.93-1.02	.	.	1.02	0.76-1.38	.	.
	BMI loss	1.04	0.97-1.12	.	.	1.37	0.86-2.17	.	.
Working hours	BMI gain	1.04	0.86-1.26	.	.	0.54	0.12-2.55	.	.
	BMI loss	1.14	0.94-1.38	.	.	1.27	0.24-6.66	.	.
*Covariates*									
Age	BMI gain	**0.71**	**0.64-0.78**	**0.71**	**0.64-0.79**	0.92	0.49-1.71	.	.
	BMI loss	**0.85**	**0.73-0.99**	0.85	0.72-1.01	0.70	0.31-1.59	.	.
Cohabitation	BMI gain	**1.29**	**1.04-1.62**	**1.33**	**1.06-1.68**	1.53	0.45-5.19	.	.
	BMI loss	0.86	0.59-1.25	0.79	0.53-1.17	0.66	0.08-5.60	.	.
Work position:									
Managers	BMI gain	0.76	0.52-1.11	.	.	**3.33**	**1.03-10.75**	.	.
	BMI loss	0.78	0.44-1.36	.	.	0.74	0.14-4.02	.	.
Others^b^	BMI gain	0.92	0.67-1.28	.	.	0.79	0.14-4.29	.	.
	BMI loss	0.62	0.35-1.10	.	.	0.39	0.04-3.52	.	.
Seniority	BMI gain	0.98	0.97-1.01	.	.	1.01	0.95-1.08	.	.
	BMI loss	1.00	0.99-1.02	.	.	0.92	0.79-1.07	.	.
Physical demands	BMI gain	1.02	0.93-1.12	.	.	0.97	0.62-1.52	.	.
	BMI loss	0.92	0.80-1.06	.	.	0.68	0.34-1.36	.	.

^a ^Significant predictors after backwards elimination.

^b ^E.g. janitors, cleaners, secretaries.

[OR = odds ratio; 95% CI = confidence intervals].

Significant results are presented in boldface.

Results from the additional analyses, where we addressed BMI as a continuous variable, showed weaker results. For women, weight change was associated with age and cohabitation, but not with any psychosocial work factors. Among men, weight change was not associated with any psychosocial work factor either (results not shown). However, we would argue that using BMI as a continuous variable could limit the possibility of finding bi-directional effects of work environment. Unsuitable weight change can occur in separate directions depending on the individual; i.e. the same work factor can increase the risk of both weight gain and weight loss.

As it is possible that the dichotomization of work hours could hide important differences between the groups, we also ran the logistic regression analyses with the original non-collapsed categories. However, regardless of this, work hours were still not predictive of weight change (results not shown).

Results from the sensitivity analyses, showed that when treated as categorical variable (three levels), quality of leadership was not a significant predictor for weight change among men. However, role clarity and role conflicts remained significant predictors, as did age and cohabitation, among women. Finally, we checked for multicollinearity by using the VIF option (Variance Inflation Factor) in the Proc Reg analysis (SAS, version 9.2). Multicollinearity, i.e. that some of the predictor variables (two or more) are highly correlated with each other, can lead to imprecise parameter estimates. The test indicated that multicollinearity was not of major concern; the highest VIF value among women was 1.87 (predictability) and among men it was 2.08 (commitment).

## Discussion

The purpose of this paper was to investigate psychosocial factors outside the classical work stress models in an attempt to determine whether these predict weight change. We hypothesized that psychosocial work factors would be associated with change in BMI when these represented an unfavourable work environment. Our hypothesis received only limited support. Among men, high leadership quality was associated with weight loss – against our expectations. Among women, we also found an unexpected association between weight change and high role clarity. In support of our hypotheses, we found that weight change (gain) was predicted by high role conflicts. Consequently, we cannot conclude that an unfavourable psychosocial work environment is associated with weight change per se.

Of the included covariates, we found age and cohabitation (among women) were associated with weight change. Specifically, living alone increased the odds for weight gain, while older age decreased the odds for weight gain. Smoking and leisure time physical activity were not included as covariates, as these can be thought of as intermediate variables in the relationship between psychosocial work environment and health outcomes. For instance, work hours may influence ones possibility for participating in leisure time physical activity, which in turn, may impact weight change.

The mixed findings of this study contribute to the existing body of literature demonstrating inconsistent results in relation to weight change and psychosocial work factors. Reviews by Overgaard and colleagues [25] and Siegrist and Rödel [55], found only modest support for a consistent relationship between work stress and bodyweight/body mass index, and Wardle and colleagues [56] concluded in a meta-analysis that work stress only has very small effect as risk factor for weight gain. Similarly, a large cohort study found that the relationship between BMI and work stress remains unclear (Nyberg et al., in press, 2011). The inconsistent findings can be a result of differences in the exposure measurement, design, or methods, but it may also be caused by a focus on an overall association. Generally, weight gain has been investigated as a function of work related stress. Two prospective studies have investigated a bi-directional relationship, and both found support for the bi-directional effects of work stress on weight [27,28]. We found some support for bi-directional effects of psychosocial work factors on weight change, in particularly with role clarity, which was associated with both BMI gain and BMI loss. Our results indicate that factors outside the classical work stress have only limited influence on weight change, some even in an unexpected direction.

Living alone was predictive of weight gain for women, but not for men. Marital status has received some attention as a factor influencing personal health, and generally, married individuals tend to weigh more than non-married [57,58]. However, some research has neglected to distinguish between cohabitating individuals and true singlehood. Making this distinction, Averett and colleagues found that cohabitation (and marriage) were associated with increased BMI [59]. Our finding did not support this; the odds of weight gain were highest among single women. Also, older age decreased the odds for weight gain.

This study had a number of strengths: the bi-directional approach to the analysis of weight change and the use of psychosocial work factors outside the traditional work stress models. First, the bi-directional strategy allows for the investigation of weight change in both directions; an approach only applied in few previous studies. Second, this study adds to prior research by using a broader mapping of the psychosocial work environment. Workload, work pace and influence at work, can be considered aspects of the job-strain model. Similarly, commitment can be considered an aspect of the effort-reward imbalance model. However, we addressed each of these as independent factors.

The assessment within a single occupational group (health care workers) provides both advantages and disadvantages, since it reduces the variation in the traditional psychosocial factors, as the workers experience similar conditions. The traditional domains of quantitative demands and influence are work factors related more to job type than to place of work. On the other hand, domains like leadership quality and predictability are much more related to the workplace than to job type. The design of the current study is optimal for detecting the latter type of effects.

Generally, our results should be interpreted in the light of the following limitations. First, our data were female-dominated; we only had 153 male participants, which limit the strength of the analyses and the generalizability. Caution must be taken when interpreting the results for men; the number of male respondents who experienced weight change was only 24. Although the attrition rate in our study was somewhat high, and potentially could cause loss of power, we would argue that bias from attrition is not a major concern in this study as we found no differences on BMI when comparing the study population with those who had only responded at baseline. Another potential limitation of this study is the fact that we conducted multiple testing. Multiple testing can be a problem as it increases the risk of mass significance. Thus, some caution must be taken when interpreting the significant results. Furthermore, we used a relatively short follow-up period (three years). A recent study among shift workers [60] found that only longer term shift work (10+ years) significantly increased the risk of obesity. This suggests that a three year follow-up period, might be insufficient to draw firm conclusions. It might be that it takes longer for the psychosocial work environment to have an impact on weight change, or that other factors (inside and outside the work environment) play a larger role in this relationship. Finally, we relied on self-reported data on weight and height, which can cause bias as height and weight are often over- and underestimated [61,62]. However, since we studied weight change, the bias is probably less than in a cross-sectional analysis.

## Conclusion

In conclusion, including alternative psychosocial work environment factors has added some new information about the relationship between work environment and weight change. Although some of these additional psychosocial work factors seem to have some effect on weight change, the associations were generally weak. Furthermore, some of the associations were in an unexpected direction. The lack of strong findings may imply that the association between weight change and work environment risk factors simply is too vague. This suggests that the risk factor model may not be the right theoretical framework for assessing the influence of the workplace on weight. Each workplace also represents a social setting where the employee interacts with co-workers (and potentially customers, clients etc.) that can set examples, provide encouragement or lack of encouragement for pursuing a healthy lifestyle. Thus, the effect of workplace on personal lifestyle might transcend the risk factors evaluated in the current study. Consequently, it is relevant to investigate the effect of workplace and to examine potential group effects within workplaces in future studies.

## Abbreviations

BMI: Body mass index; COPSOQ: Copenhagen Psychosocial Questionnaire; OR: Odds ratio; CI: Confidence interval.

## Competing interests

The authors declare that they have no competing interests.

## Authors’ contributions

HGQ has developed the idea for this article and was responsible for the development of the design, data processing and the statistics analyses. Furthermore, HGQ has interpreted the results, written the manuscript and prepared it for publication. UC has contributed substantially to the development of the design, interpretation of the results and provided critical revisions to the manuscript. KBC has contributed to the development of the design and the statistical analyses. BA has provided critical revisions to the manuscript and to the design of the study. VB has provided critical revisions to the interpretation of the results and the design of the study. JBB has supervised the entire process and significantly contributed to the design, statistical analyses, interpretation of the results and made critical revisions to the manuscript. All authors have read and approved the manuscript in its final form.

## Pre-publication history

The pre-publication history for this paper can be accessed here:

http://www.biomedcentral.com/1471-2458/13/43/prepub
